# In the article “An overview of the implementation of the first doctorate program in nursing in the Northern region”

**DOI:** 10.1590/0034-7167.20257804e04

**Published:** 2025-09-01

Where it read:

Line 1: The State University of Pará (UEPA) has just (...)

Line 17: The doctorate program in nursing at the State University of Pará is designed to (...)

Line 23: UEPA has brought together professionals (...)

Line 39: ... to follow UEPA’s example and expand the provision of advanced (...)

Line 42: ... by the State University of Pará is a milestone for education (...)

Line 44: ... but also reinforces UEPA’s commitment to excellence (...)

It reads:

Line 1: The State University of Pará (UEPA) and the Federal University of Amazonas (UFAM) have just (...)

Line 17: The doctorate program in nursing at the UEPA and UFAM are designed to (...)

Line 23: UEPA and UFAM has brought together professionals (...)

Line 39: ... to follow UEPA and UFAM’s example and expand the provision of advanced (...)

Line 42: ... by the State University of Pará and the Federal University of Amazonas are a milestone for education (...)

Line 44: ... but also reinforces UEPA and UFAM’s commitment to excellence (...)

