# Supplementary material for: In the article “An overview of the implementation of the first doctorate program in nursing in the Northern region”
Source: Rev Bras Enferm. 2025 Sep 1;78(4):e2025n4e04. doi: 10.1590/0034-7167.20257804e04 (PMC12407868; doi:10.1590/0034-7167.20257804e04)
Supplement: Related texts

# Correction: No artigo “O olhar sobre a implantação do primeiro doutorado em enfermagem da Região Norte”,

**DOI:** 10.1590/0034-7167.20257804e04pt

Onde se lia:

Linha 1: A Universidade do Estado do Pará (UEPA) acaba de (...)

Linha 18: O doutorado em Enfermagem da UEPA foi (...)

Linha 25: A UEPA reuniu profissionais (...)

Linha 43: ... da UEPA e a expandir a oferta (...)

Linha 45: ... pela Universidade do Estado do Pará é um marco (...)

Linha 48: ... compromisso da UEPA com a excelência (...)

Lê-se:

Linha 1: A Universidade do Estado do Pará (UEPA) e a Universidade Federal do Amazonas (UFAM) acabam de (...)

Linha 18: O doutorado em Enfermagem da UEPA e da UFAM foi (...)

Linha 25: A UEPA e a UFAM reuniram profissionais (...)

Linha 43: ... da UEPA e UFAM e a expandir a oferta (...)

Linha 45: ... pela UEPA e UFAM é um marco (...)

Linha 48: ... compromisso da UEPA e da UFAM com a excelência (...)

# Correction: Un vistazo a la implementación del primer doctorado en enfermería en la región Norte

**DOI:** 10.1590/0034-7167.20257804e04es

Donde decía:

Línea 1: La Universidad Estatal de Pará (UEPA) acaba de (...)

Línea 18: El Doctorado en Enfermería de la UEPA ha sido (...)

Línea 25: UEPA ha reunido a profesionales (...)

Línea 43: ...de UEPA y a extender la oferta (...)

Línea 46: ...en la Universidad del Estado de Pará constituye un hito (...)

Línea 49: ...el compromiso de la UEPA con la excelencia (...)

Dice:

Línea 1: La Universidad Estatal de Pará (UEPA) y la Universidad Federal del Amazonas (UFAM) acaban de (...)

Línea 18: El Doctorado en Enfermería por la UEPA y UFAM han (...)

Línea 25: UEPA y UFAM han reunido profesionales (...)

Línea 43: ...de la UEPA y de la UFAM y a extender la oferta (...)

Línea 46: ... por UEPA y UFAM constituyen un hito (...)

Línea 49: ...compromiso de la UEPA y de la UFAM con la excelencia (...)
